# Improving Intensity-Based Lung CT Registration Accuracy Utilizing Vascular Information

**DOI:** 10.1155/2012/285136

**Published:** 2012-11-28

**Authors:** Kunlin Cao, Kai Ding, Joseph M. Reinhardt, Gary E. Christensen

**Affiliations:** ^1^Biomedical Image Analysis Lab, GE Global Research Center, Niskayuna, NY 12309, USA; ^2^Department of Radiation Oncology, Virginia Commonwealth University, Richmond, VA 23298, USA; ^3^Department of Biomedical Engineering, The University of Iowa, Iowa City, IA 52242, USA; ^4^Department of Electrical and Computer Engineering, The University of Iowa, Iowa City, IA 52242, USA; ^5^Department of Radiation Oncology, The University of Iowa, Iowa City, IA 52242, USA

## Abstract

Accurate pulmonary image registration is a challenging problem when the lungs have a deformation with large distance. In this work, we present a nonrigid volumetric registration algorithm to track lung motion between a pair of intrasubject CT images acquired at different inflation levels and introduce a new vesselness similarity cost that improves intensity-only registration. Volumetric CT datasets from six human subjects were used in this study. The performance of four intensity-only registration algorithms was compared with and without adding the vesselness similarity cost function. Matching accuracy was evaluated using landmarks, vessel tree, and fissure planes. The Jacobian determinant of the transformation was used to reveal the deformation pattern of local parenchymal tissue. The average matching error for intensity-only registration methods was on the order of 1 mm at landmarks and 1.5 mm on fissure planes. After adding the vesselness preserving cost function, the landmark and fissure positioning errors decreased approximately by 25% and 30%, respectively. The vesselness cost function effectively helped improve the registration accuracy in regions near thoracic cage and near the diaphragm for all the intensity-only registration algorithms tested and also helped produce more consistent and more reliable patterns of regional tissue deformation.

## 1. Introduction

 Image registration is used to find the spatial correspondence between two images and plays an important role in pulmonary image analysis. In a sequence of pulmonary scans, image registration provides the spatial locations of corresponding voxels. The computed correspondences describe the motion of the lung between a pair of images at the voxel level. Registration of lung volumes across time or across modalities has been utilized to establish lung atlases [1], estimate regional ventilation and local lung tissue expansion [2–5], assess lobar slippage during respiration [6, 7], and measure pulmonary function change following radiation therapy [8].

Lung registration methods are typically intensity-based [2, 5, 9–13] or feature-based [14–18]. Intensity-based methods consider intensity patterns of the whole lung regions to define similarity measures. They take advantage of the strong contrast between the lung parenchyma and the chest wall, and between the parenchyma, the blood vessels and larger airways. Commonly used intensity-based methods include minimizing intensity difference [3, 10], maximizing mutual information or normalized cross correlation [5, 9], and preserving tissue volume or lung mass [12, 13]. Since intensity-based methods do not use anatomical knowledge, these methods can get stuck in local minima resulting in mismatches of important anatomical structures such as bifurcations of smaller airway and vessel branches. On the other hand, feature-based methods define transformations utilizing corresponding features derived from original images. They usually utilize corresponding landmarks and local intensity patterns [14, 16, 17] and surfaces correspondences [14, 15, 18]. However, due to the sparsity of features, good alignment of features can not guarantee satisfactory matching accuracy for all lung regions.

Since registration methods using either intensity-only or feature-only registration have their limitations, it is desirable to design lung registration methods that utilize both intensity and feature information [19–23]. It has been shown that hybrid registration methods that combine intensity and feature information can help improve matching accuracy. Most of these methods incorporate distributed landmark pairs selected at airway or vessel branch points, that were identified manually or semiautomatically [24], centerline of the airway and vessel tree structures, and lung surface information. These feature extractions may be difficult and time-consuming tasks. Therefore, fast feature extractions and effective methods to utilize feature information are needed to improve registration accuracy.

In this paper, we couple intensity and feature information together to match 3D lung CT images acquired during breath hold at two different levels of inflation. We propose a feature-based similarity criterion utilizing the information of vessel locations and shapes in the registration process. This vesselness preserving cost function is added to four existing intensity-based similarity costs, and comparison experiments show that this criterion helps improve the registration accuracy. Higher matching accuracy makes the postanalysis of regional tissue mechanical properties more plausible and reliable.

Our preliminary work on the effectiveness of using a vesselness matching similarity term was described in [25–27]. The work presented in this paper provides a complete description of our vesselness matching approach. This paper extends our previous work by describing how to choose weighting factors for the different cost terms, describes a multiresolution optimization scheme, and provides more validation. This paper studies the effect of the vessel matching when used with various intensity similarity metrics such as the sum of squared intensity difference, sum of squared tissue volume difference, mutual information, and normalized cross correlation. This paper also examines the effect of using a linear-elastic constraint to regularize the displacement fields.

## 2. Material and Methods

### 2.1. Image Data Sets

**Figure 1:**
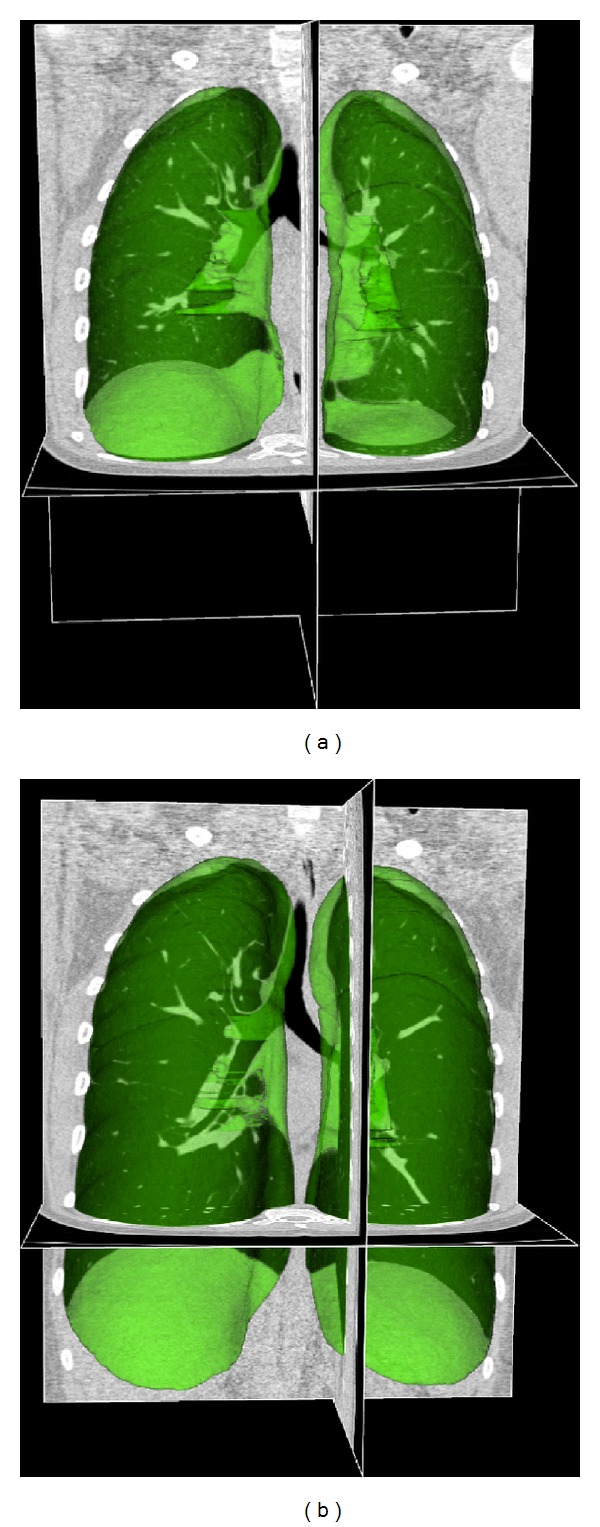
Pulmonary CT images acquired at breath hold (a) maximum exhale and (b) maximum inhale with renderings of the lung segmentations (green objects).

 In this study, six pairs of volumetric CT data sets from six human subjects in the supine orientation were collected on a Siemens Sensation 64 multidetector CT scanner. Each image pair was acquired during breath-holds near functional residual capacity (FRC) and total lung capacity (TLC) in the same scanning session. For subject 1, data were acquired at functional residual capacity (FRC) with 21.8% of the vital capacity (VC) and total lung capacity (TLC) with 95.6% of the VC. For subject 2, data were acquired at FRC with 30.5% of the VC and TLC with 89.6% of the VC. For subject 3, data were acquired at FRC with 26.3% of the VC and TLC with 95.7% of the VC. For subject 4, data were acquired at FRC with 11.0% of the VC and TLC with 68.9% of the VC. For subject 5, data were acquired at FRC with 25.8% of the VC and TLC with 92.9% of the VC. For subject 6, data were acquired at FRC with 26.5% of the VC and TLC with 102.0% of the VC. Each volumetric data set was acquired at a section spacing of 0.5~0.6 mm and a reconstruction matrix of 512 × 512. In-plane pixel spatial resolution was approximately 0.6 mm × 0.6 mm. The parenchyma regions in the FRC and TLC data sets were segmented using the method described in [28].  Figure 1 gives an illustration of pulmonary CT images with renderings of the lung segmentations.

### 2.2. Image Registration

The goal of image registration is to estimate a transformation *h* that defines the pointwise correspondences between two images *I*_1_ and *I*_2_. More formally, let *D* ⊂ *R* ^3^ define the domain of the images *I*_1_ : *D* → *R* and *I*_2_ : *D* → *R*. In this work, the transformation *h* : *D* → *D* is assumed to be a diffeomorphism. Let *ℋ* denote the set of all diffeomorphisms from *D* to *D*. The optimal transformation *h* ∈ *ℋ* is estimated by minimizing a cost function that consists of a similarity cost function and a regularizing term, that is,

(1) $$\begin{array}{c} \hat{h}=\underset{h\in ℋ}{\text{argmin}}C_{\text{SIM}}\left(I_{1},I_{2},h\right)+\lambda C_{\text{REG}}\left(h\right). \end{array}$$

The similarity cost function describes the characteristics of two images that should agree for corresponding image points. For example, these characteristics often correspond to matching intensity characteristics and features. The regularization cost function is used to enforce desired properties of the transformation such as minimum distortion. The constant *λ* is used to balance the influence of the regularization cost with respect to the similarity cost. In general, the similarity and the regularization costs can be decomposed into linear combinations of more specific cost functions.

#### 2.2.1. Intensity-Based Similarity Cost Functions

 For intensity-based image registration, it is usually assumed that intensities of corresponding voxels are related to each other in some way. Many criteria to construct the intensity relationship between corresponding points have been suggested as the cost function for aligning two images. Examples of intensity-based cost functions are the mean square difference (MSD), correlation coefficient, mutual information, pattern intensity, and gradient correlation [29, 30]. In this work, we investigated three commonly used intensity-based cost functions and one intensity-based cost function designed for matching lung CT images.

**Sum of Squared Difference (SSD)**

Minimizing the intensity difference at corresponding points between two images is an intuitive method to register grayscale images. A simple and common cost function is the sum of squared difference (SSD) defined by

(2) $$\begin{array}{c} C_{\text{SSD}}=\sum_{x\in \Omega }{\left[I_{2}(x)-I_{1}(h(x))\right]}^{2}, \end{array}$$

where *I*_1_ and *I*_2_ are the template and target image intensity functions, respectively. **Ω** ⊂ *R* ^3^ denotes the union of lung regions in target image and deformed template image. The underlying assumption of SSD is that the image intensity at corresponding points between two images should be similar. This is true when registering images of the same modality. However, considering the change in CT intensity as air inspired and expired during the respiratory cycle, the grayscale range is different within the lung region in two CT images acquired at different inflation levels. To balance this grayscale range difference, intensity normalization is needed. For example, a histogram matching procedure [31] can be used before SSD registration to modify the histogram of template image so that it is similar to that of target image.

**Mutual Information (MI)**

Mutual information (MI) similarity cost function can accommodate intensity difference between two images and is therefore well-suited to accommodate the CT intensity change during inflation and deflation of the lung. Mutual information expresses the amount of information that one image contains about the other one. Analogous to the Kullback-Leibler measure, the negative mutual information cost of two images is defined as [9, 32]

(3) $$\begin{array}{c} C_{\text{MI}}=-\sum_{i}\sum_{j}p\left(i,j\right)\log\frac{p\left(i,j\right)}{p_{I_{1}\circ h}\left(i\right)p_{I_{2}}\left(j\right)}, \end{array}$$

where *p*(*i*, *j*) is the joint intensity distribution of transformed template image *I*_1_∘*h* and target image *I*_2_; *p*_*I*_1_∘*h*_(*i*) and *p*_*I*_2__(*j*) are their marginal distributions, respectively. The histogram bins of *I*_1_∘*h* and *I*_2_ are indexed by *i* and *j*. The experiments of MI-driven registration use 50 × 50 histogram bins to estimate joint distribution.

**Normalized Cross Correlation (NCC)**

Normalized cross correlation can be used for multimodality registration problems since it is insensitive to a constant multiplicative factor between the images. This cost function measures the pixel-wise cross-correlation between image intensities normalized by the square root of the autocorrelation of each image. Mathematically, the negative normalized cross correlation measure is given by [33]

(4) $$\begin{array}{c} C_{\text{NCC}}=-\frac{\sum_{x\in \Omega }I_{2}\left(x\right)\cdot I_{1}\left(h\left(x\right)\right)}{\sqrt{\sum_{x\in \Omega }I_{2}{\left(x\right)}^{2}\cdot \sum_{x\in \Omega }I_{1}{\left(h\left(x\right)\right)}^{2}}}, \end{array}$$

where the negative sign was added so that the optimal transformation **h** is found by minimization. When the factor of the intensity patterns from two images is a constant, the measure equals −1. Misalignments between the images will result in decrease of the normalized cross correlation, and thus, increase of the similarity cost *C*_NCC_.

**Sum of Squared Tissue Volume Difference (SSTVD)**

The sum of squared tissue volume difference (SSTVD) cost function [13] accounts for the variation of intensity in the lung CT images during respiration. Assume that lung is a mixture of two materials: air and tissue/blood (nonair). Then the Hounsfield units (HU) in lung CT images is a function of tissue and air content. From the HU of CT lung images, the regional tissue volume and air volume can be estimated following the air-tissue mixture model by Hoffman and Ritman [34]. Let *v*(**x**) be the volume element at location **x**. Then the tissue volume *V*(**x**) within the volume element can be estimated as *V*(**x**) = *v*(**x**)((HU(**x**) − HU_air_)/(HU_tissue_ − HU_air_)), where we assume that HU_air_ = −1000 and HU_tissue_ = 0. The intensity similarity cost function SSTVD is defined as [13]

(5) CSSTVD=∫Ω[V2(x)−V1(h(x))]2dx =∫Ω[v2(x)I2(x)+10001055  −v1(h(x))I1(h(x))+10001055]2dx.

The Jacobian of a transformation *J*(**h**) estimates the regional volume changes resulted from mapping corresponding regions. Thus, the tissue volumes in image *I*_1_ and *I*_2_ are related by *v*_1_(**h**(**x**)) = *v*_2_(**x**) · *J*(**h**(**x**)).

#### 2.2.2. Feature-Based Similarity Cost Function

Feature information extracted from the grayscale image is important to help guide the registration process. During the respiration cycle, blood vessels keep their tubular shapes and tree structures. Therefore, the shape and spatial information of vessels can be utilized to help improve the registration accuracy. In CT images, blood vessels have larger intensity values than that of parenchyma tissues. This intensity difference between parenchyma and blood vessels can effectively help intensity-based registration. However, the diameter of vessel becomes smaller as the blood vessel branches. The small blood vessels are difficult to visualize because of their low intensity contrast. Therefore, grayscale information of the small vessels give almost no contribution to the intensity-based registration. In order to better utilize the information of blood vessel locations, we utilize the vesselness measure (VM) computed from intensity images rather than using their grayscales directly.

**Sum of Squared Vesselness Measure Difference (SSVMD)**

The vesselness measure is based on the analysis of eigenvalues of the Hessian matrix of image intensity. The eigenvalues, which are geometrically interpreted as principal curvatures, can be used to indicate the shape of underlying object. In 3D lung CT images, isotropic structures such as parenchyma tissues (dark) are associated with three similar nonzero positive eigenvalues. Tubular structures such as blood vessels (bright) are associated with one negligible eigenvalue and two similar nonzero negative eigenvalues [35]. Ordering the eigenvalues of a Hessian matrix by magnitude |*λ*_1_ | ≤|*λ*_2_ | ≤|*λ*_3_|, the Frangi's vesselness function [35] is defined as

(6) F(λ)={(1−e−RA2/2α2)·e−RB2/2β2·(1−e−S2/2ρ2)         if  λ2<0  and  λ3<0,0              otherwise,

with $R_{A}=\left|\lambda_{2}\right|/\left|\lambda_{3}\right|,R_{B}=\left|\lambda_{1}\right|/\sqrt{\left|\lambda_{2}\lambda_{3}\right|},S=\sqrt{\lambda_{1}^{2}+\lambda_{2}^{2}+\lambda_{3}^{2}}$, where *R*_*A*_ distinguishes between plate-like and tubular structures, *R*_*B*_ accounts for the deviation from a blob-like structure, and *S* differentiates between tubular structures and noise. *α*, *β*, and *ρ* control the sensitivity of the vesselness measure. The experiments in this paper use *α* = 0.5, *β* = 0.5, and *ρ* = 5.

The Hessian matrix is computed by convolving the intensity image with second and cross derivatives of the Gaussian function. In a multiscale analysis, the response of the vesselness filter will achieve the maximum at a scale, which approximately matches the size of vessels to detect. Therefore, the vesselness measure is estimated by computing (6) for a range of scales and selecting the maximum response: *F* = max⁡_*σ*_min⁡_≤*σ*≤*σ*_max⁡__ *F*(*λ*), where *σ* is the standard deviation of the Gaussian function [36].

**Figure 2:**
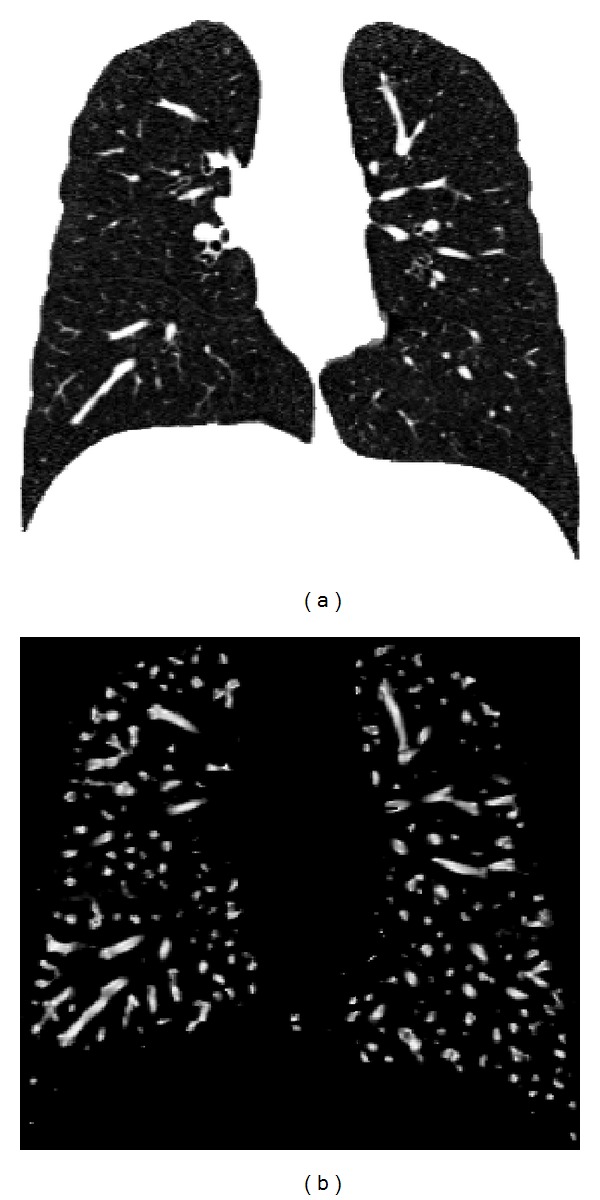
The vesselness images calculated from lung CT grayscale images. (a) A coronal slice of TLC data and (b) its vesselness measure. Vesselness measure is computed in multiscale analysis with *σ* = [2,3] and rescaled to [0, 1].

The vesselness image is rescaled to [0, 1] and can be considered as a probability-like estimate of vesselness features. Larger vesselness value indicates that the underlying object is more likely to be a vessel structure, as shown in Figure 2. Let *F*_1_(**x**) and *F*_2_(**x**) represent the vesselness measures of images *I*_1_ and *I*_2_ at location **x**, respectively. In order to match similar vesselness patterns between two images, the sum of squared vesselness measure difference (SSVMD) is proposed as

(7) $$\begin{array}{c} C_{\text{SSVMD}}=\sum_{x\in \Omega }{\left[F_{2}(x)-F_{1}(h(x))\right]}^{2}. \end{array}$$

Mismatch from vessel to tissue structures will result in a larger SSVMD cost.

#### 2.2.3. Elastic Regularization

 Enforcing constraints on the transformation helps generate physiologically more meaningful registration results. Continuum mechanical models such as linear elasticity [11, 37, 38] and viscous fluid [37, 39] can be used to regularize the transformation. A common way to constraint the deformation is applying a differential operator on the transformation and formulating an additive cost term in the objective cost function [11, 22, 40–46]. In our registration algorithms, a linear-elastic constraint was used to regularize the displacement fields **u**, where **u** = **h**(**x**) − **x**. This regularization term is formed as

(8) $$\begin{array}{c} C_{\text{REG}}\left(u\right)=\int_{\Omega }{||Lu\left(x\right)||}^{2}dx, \end{array}$$

where *L* can be any nonsingular linear differential operator [47]. Here the linear elasticity operator *L* is formed as *L* **u**(**x**) = −*α*∇^2^ **u**(**x**) − *β*∇(∇·**u**(**x**)) + *γ* **u**(**x**), where ∇ = [∂/∂*x*_1_, ∂/∂*x*_2_, ∂/∂*x*_3_] and ∇^2^ = ∇·∇ = [∂^2^/∂*x*_1_^2^ + ∂^2^/∂*x*_2_^2^ + ∂^2^/∂*x*_3_^2^].

Using the linear elasticity differential operator can help smooth the transformation and help eliminate abrupt changes in the displacement fields. The linear elasticity operator is used in this work to help avoid the transformation from folding onto itself. However, it cannot prevent the Jacobian of the transformation from going negative, that is, destroying the image topology under the transformation [48]. Additional constraints on the displacement parameters are applied in the optimization method.

#### 2.2.4. Total Cost Function

 Finally, the total cost is defined as a linear combination of the intensity-based costs, vesselness measure preserving cost, and regularization constraint

(9) CTOTAL(h)=CINTENSITY(I1,I2,h)+χCSSVMD(I1,I2,h)+γCREG(h).

*C*_INTENSITY_ can be one of the four intensity-based similarity cost functions: *C*_SSD_, *C*_MI_, *C*_NCC_, or *C*_SSTVD_. Constants *χ* and *γ* are weights to adjust the significance of the three terms.

### 2.3. Transformation Parameterization

 The transform defines how points from the template image *I*_1_ are mapping to their corresponding points in the target image *I*_2_. In three dimensional space, let **x** = (*x*_1_, *x*_2_, *x*_3_)^*T*^ define a voxel coordinate in the image domain of the target image *I*_2_. The transformation **h** is a (3 × 1) vector-valued function defined on the voxel lattice of target image, and **h**(**x**) gives the corresponding location in template image to the point **x**.

The lung is composed of nonhomogenous soft tissue. Lung tissue expansion varies in different lung regions. Since lung expansion is nonuniform, nonrigid transformations are required to model the lung motion across different inflation levels. To represent the locally varying geometric distortions, the transformation can be represented by various forms of basis function, such as Fourier transform, thin-plate splines, and B-splines. B-splines are well suited for image registration and are able to capture the local nonrigid deformation between two images [40, 45]. Considering the computational efficiency and accuracy requirement, the cubic B-spline based parameterization was chosen to represent the transformation.

Let *ϕ*_*i*_ = [*ϕ*_*x*_(**x**_*i*_), *ϕ*_*y*_(**x**_*i*_), *ϕ*_*z*_(**x**_*i*_)]^*T*^ be the coefficients of the *i*th control point **x**_*i*_ on the spline lattice *G* along each direction. The transformation is represented as

(10) $$\begin{array}{c} h\left(x\right)=x+\sum_{i\in G}\phi_{i}\beta \left(x-x_{i}\right), \end{array}$$

where *β*(*x*, *y*, *z*) = *β*(*x*)*β*(*y*)*β*(*z*) is a separable convolution kernel. *β*(*x*) is the uniform cubic B-spline basis function defined as

(11) $$\begin{array}{c} \beta \left(x\right)=\{\begin{array}{c} \frac{\left(4-6x^{2}+3{\left|x\right|}^{3}\right)}{6},\;0\leq \left|x\right|<1\text{,}\\ \frac{{\left(2-\left|x\right|\right)}^{3}}{6},\;\;\;\;\;1\leq \left|x\right|<2\text{,}\\ 0,\;\;\;\;\;\;\;\;\;\;\;\;\left|x\right|\geq 2. \end{array} \end{array}$$

### 2.4. Optimization Method

Most registration cost functions can be minimized using standard optimization techniques. There are several existing methods in numerical analysis such as the partial differential equation (PDE) solvers used to solve for elastic and fluid transformations, steepest gradient descent method, conjugate gradient method, and so forth. Similar to [9, 13], our similarity cost functions were optimized using a limited memory quasi-Newton minimization method with bounds (L-BFGS-B) [49] algorithm. It is well suited for optimization with a high-dimensional parameter space. In addition, this algorithm allows bound constraints on the independent variables.

The bound constraints are applied on B-Spline coefficients to guarantee the local injectivity (one-to-one property) of the transformation [50], that is, the transformation maintains the topology of two images. According to their analysis, the displacement fields are locally injective over the domain if the B-Spline coefficients satisfy the conditions that *ϕ*_*x*_ ≤ *δ*_*x*_/*K*, *ϕ*_*y*_ ≤ *δ*_*y*_/*K*, and *ϕ*_*z*_ ≤ *δ*_*z*_/*K*, where *δ*_*x*_, *δ*_*y*_, and *δ*_*z*_ are the B-Spline grid sizes along each direction, and *K* is a constant approximately equal to 2.479772335.

### 2.5. Registration Accuracy Assessment

 Validation and evaluation of image registration accuracy is an important task to quantify the performance of registration algorithms. Due to the absence of a “gold standard” to judge a registration algorithm, multiple evaluation methods are needed to evaluate the performance of image registration algorithms.

#### 2.5.1. Landmark Matching Accuracy

**Figure 3:**
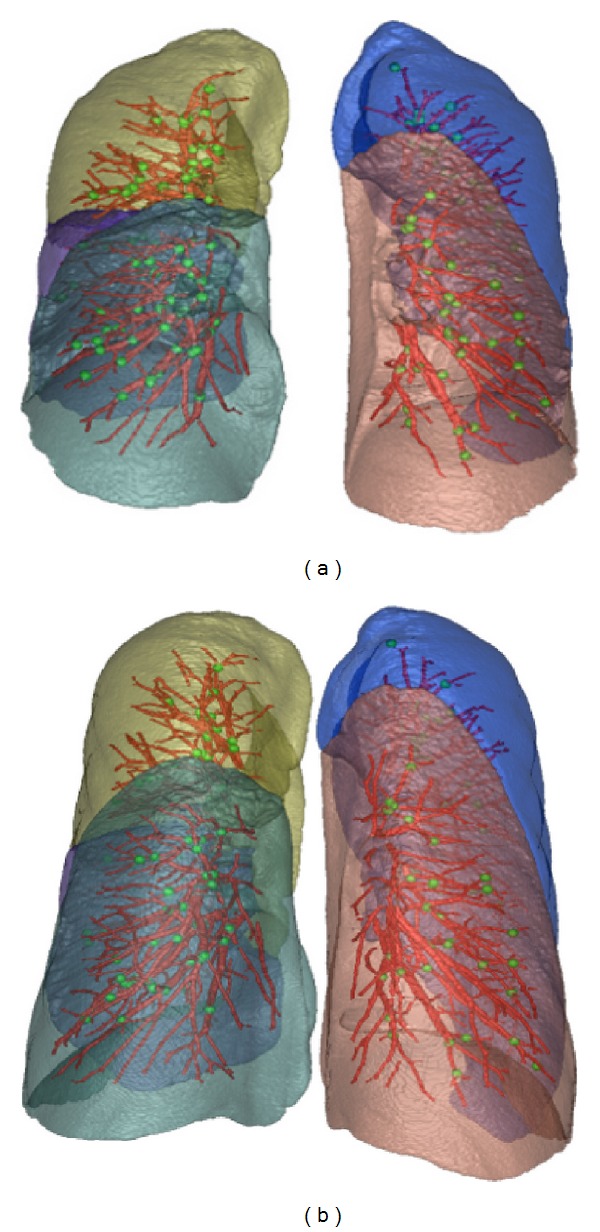
Distribution of landmarks (green points) selected at vessel-tree branch points on (a) FRC and (b) TLC scans of one subject. Vessel trees are marked as red curves. Different lobes are marked using different colors.

 Intrasubject CT images of the lung contain identifiable landmarks such as airway-tree and vascular-tree branch points. For each pair of FRC and TLC data, 100–150 distinctive landmark pairs were selected as branch points of the vascular tree using a semiautomatic method [24]. The landmarks were selected so that they were dispersed throughout the lungs. An example of the landmark distribution is shown as green points in Figure 3. The Euclidean distance between the registration-predicted landmark position and its true position is defined as landmark error. Let **p**_*k*_ and **q**_*k*_ be the location of landmark *k* on template image *I*_1_ and target image *I*_2_, respectively. The landmark error is calculated as *d* = ||**p**_*k*_ − **h**(**q**_*k*_)||. 

#### 2.5.2. Vessel Matching Accuracy

 Vessels in the lung keep their tubular shape and tree structures during the respiratory process. The vascular tree provides rich spatial and shape information in parenchyma regions. Therefore, evaluating the alignment on vessel trees is an important approach to validate the matching accuracy at the lung feature level.

The registration accuracy on the vessel tree was evaluated by vessel matching distance, which is calculated as the distance between a point on the target vessel tree and its closet point on warped template vessel tree. Mathematically, this distance can be stated as the vessel positioning error (VPE)

(12) $$\begin{array}{c} \text{VPE}\left(x\right)=\underset{y\in V_{2}}{\min}\;d\left(x,h\left(y\right)\right) \end{array}$$

for a given point **x** in *V*_1_, where *V*_1_ and *V*_2_, respectively, are the set of all points in the vessel trees extracted from image *I*_1_ and *I*_2_, respectively, and *d*(·) defines the Euclidean distance. The vessel positions used for validation are segmented using vessel segmentation algorithm [51]. Examples of the vessel tree extractions are shown as red curves in Figure 3.

#### 2.5.3. Fissure Alignment Distance

 The human lungs are divided into five independent compartments called lobes. Lobar fissures are defined as the division between adjacent lung lobes and represent important physical boundaries within the lungs. Therefore, alignment of fissures is an important evaluation criterion. Fissure locations are extracted from the images by segmenting the lobes using [52] and then identifying voxels adjacent to two lobe segmentations. The fissure positioning error (FPE) is defined as the minimum distance between a point on the deformed fissure and the closest point on the corresponding target fissure

(13) $$\begin{array}{c} \text{FPE}\left(x\right)=\underset{y\in F_{2}}{\min}\;d\left(x,h\left(y\right)\right) \end{array}$$

for a given point **x** in *F*_1_, where *F*_1_ and *F*_2_, respectively, are the set of all points in the fissure in image *I*_1_ and *I*_2_, respectively. Examples of the lobe segmentations are shown in different colors in Figure 3.

#### 2.5.4. Assessment of Lung Function by the Jacobian Determinane

 The lung tissue deformation pattern can be used as an index to assess lung function. In this work, the Jacobian determinant of the transformation field derived by image registration is used to estimate the local tissue deformation [53].

The Jacobian determinant (often simply called the Jacobian) [11, 48, 54] is a measurement to estimate the pointwise expansion and contraction during the deformation. The Jacobian of the transformation *J*(**h**(**x**)) is defined as

(14) $$\begin{array}{c} J\left(h\left(x\right)\right)=\left|\begin{array}{ccc} \frac{\partial h_{1}\left(x\right)}{\partial x_{1}} & \frac{\partial h_{1}\left(x\right)}{\partial x_{2}} & \frac{\partial h_{1}\left(x\right)}{\partial x_{3}}\\ \frac{\partial h_{2}\left(x\right)}{\partial x_{1}} & \frac{\partial h_{2}\left(x\right)}{\partial x_{2}} & \frac{\partial h_{2}\left(x\right)}{\partial x_{3}}\\ \frac{\partial h_{3}\left(x\right)}{\partial x_{1}} & \frac{\partial h_{3}\left(x\right)}{\partial x_{2}} & \frac{\partial h_{3}\left(x\right)}{\partial x_{3}} \end{array}\right|. \end{array}$$

Using a Lagrangian reference frame, a Jacobian value of one corresponds to zero expansion or contraction. Local tissue expansion corresponds to a Jacobian greater than one, and local tissue contraction corresponds to a Jacobian less than one.

### 2.6. Preprocessing

 Preprocessing starts by identifying the lung regions in all images using the method described in [28]. Images and masks are downsampled by a factor of 2 in each dimension to reduce computation time. For each pair of data sets, FRC images are used as the target image, and TLC images are used as the template image.

In order to evaluate how the vesselness cost function affects the registration algorithm results, we performed registration experiments using different similarity costs on parenchyma region in each pair of data sets for comparison. There were four registration methods driven by intensity-only similarity cost functions described in Section 2.2.1, and the same four registration methods that included the feature-based similarity cost SSVMD. After registration, the results from each method were evaluated and compared through matching accuracy on landmarks, vessels, fissures, and underlying transformation properties.

## 3. Results 

### 3.1. Tuning Weighing Parameters

 We designed experiments to discover good parameter settings on intensity-based cost, feature-based cost *C*_SSVMD_, and regularization term *C*_REG_ in (9). Here we discuss our approach for selecting registration parameters for *C*_SSTVD_. Parameter settings for registration algorithms using other intensity-based cost functions, for example, *C*_SSD_, *C*_MI_, and *C*_NCC_ can be tuned in the same way.

**Figure 4:**
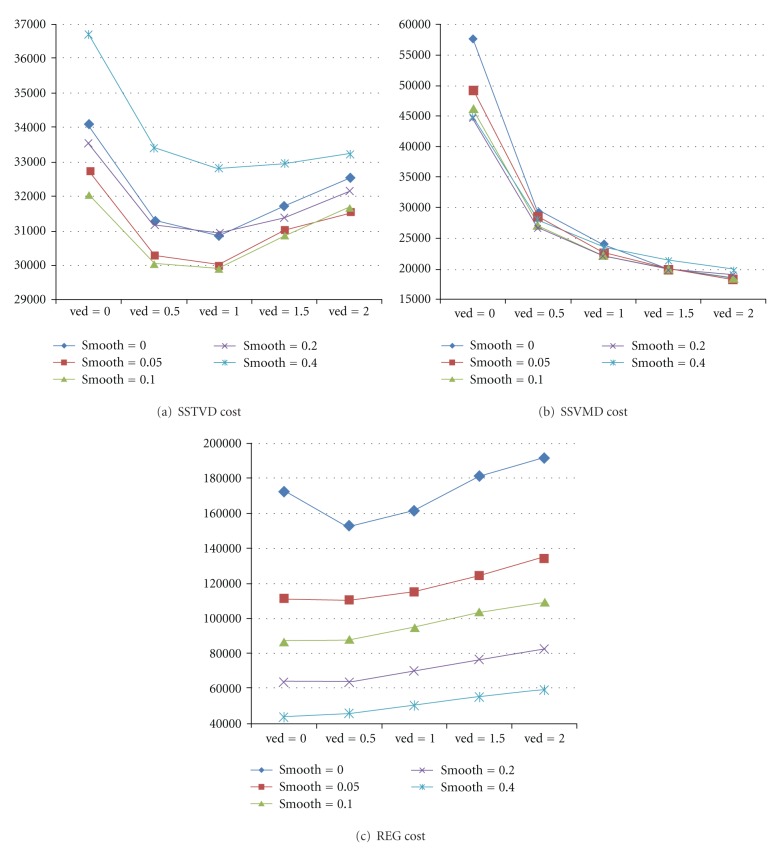
Plots of (a) SSTVD, (b) SSVMD, and (c) REG costs using different parameter settings. Data are averaged through results from six subjects.

Table 1 and Figure 4 show the results for 20 CT-to-CT registration experiments, as the weighting values *χ* (ved) and *γ* (smooth) are varied. The cost functions were values averaged on the results from six subjects. The values of *χ* and *γ* ranged from 0 to 2 and 0 to 0.5, respectively.

Experiment CT01 corresponds to unconstrained estimation, in which the transformation was estimated only according to the intensity similarity cost. This experiment produced relatively the worse registration results as evident by the large values of *C*_SSTVD_, *C*_SSVMD_, and *C*_REG_ in the respective tables.

Experiments CT02, CT03, CT04, and CT05 demonstrate the effect of estimating the transformations without minimizing the vesselness similarity cost while varying *γ* the weight of the linear elastic cost. Minimizing the linear elastic cost is good for optimizing the other two similarity costs *C*_SSTVD_ and *C*_SSVMD_, as we can see from Figures 4(a) and 4(b). *γ* values larger than 0.2 are not recommended since they may cause the *C*_SSTVD_ to increase dramatically. Increasing the constraint weights results in a worse intensity match between images.

Experiments CT06, CT11, CT16, and CT21 demonstrate the effect of using vesselness similarity cost function without enforcing the linear elasticity constraint. The *C*_SSVMD_ values for these experiments are much lower than the previous cases since they are being minimized. The intensity similarity costs *C*_SSTVD_ also decreased using registration with vesselness constraint, especially when *χ* is in the range of [0.5, 1].

The remaining experiments show the effect of jointly estimating the transformations, while varying the weights on both the vesselness similarity cost and the linear elasticity constraint. These experiments show that it is possible to find a set of parameters that produce better results using both constraints than only using one or none.

The optimal set of parameters should be chosen to provide a good intensity match and vesselness match, while producing less spatial distortion as measured by an acceptable level of linear elastic cost. From the experiments, we observe that *χ* = 0.5 ~ 1 and *γ* = 0.05 ~ 0.1 are good for minimizing the three costs at the same time. In this work, weighting parameters were set to *χ* = 1 and *γ* = 0.1 when using SSTVD as intensity cost function. Parameters in registration using other three intensity cost functions are set in the same way.

### 3.2. Multiresolution Optimization Scheme

A spatial multiresolution optimization procedure from coarse to fine was used to improve speed, accuracy, and robustness of the registration. In the experiments, the spatial multiresolution strategy proceeds from low to high resolution, starts at one-eighth of the spatial resolution, and increases by a factor of two until the full resolution is reached. Meanwhile, a hierarchy of B-Spline grid spacings from large to small was also used. The finest B-spline grid space used in the experiments was 4 mm. An example multiresolution scheme design for minimizing the total cost function is listed in Table 2. The image spatial resolution and B-spline grid spacing were refined alternatively.

**Figure 5:**
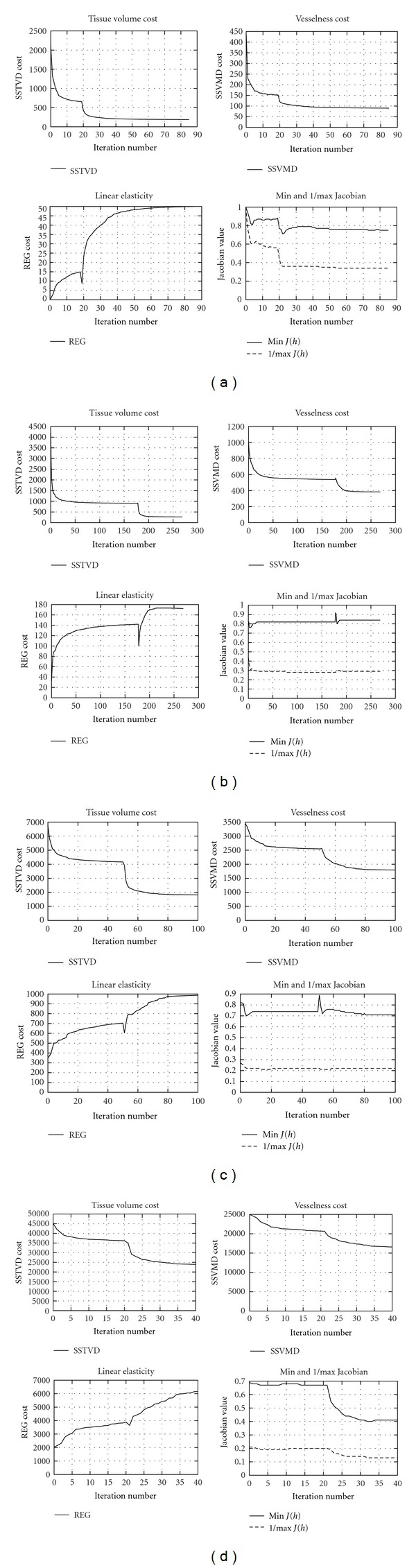
Cost functions versus iteration associated with experiment CT13. Cost function values are recorded at (a) 1/8, (b) 1/4, (c) 1/2, and (d) full image resolution.

Figure 5 lists the cost values at each iteration of one registration. At 1/8 and 1/4 image resolutions, registration speed is fast and runs for more iterations. The optimization is stopped before reaching the maximum iterations if the total cost change is nominal between consecutive iterations. Global shapes are matched at these two levels. At 1/2 image resolution, the inner structures get clearer and are aligned roughly. Registration at full resolution further adjusts the local structures matching. During the registration procedure, the Jacobian values are checked to make sure that they remain positive.

### 3.3. Landmark Matching Accuracy

**Figure 6:**
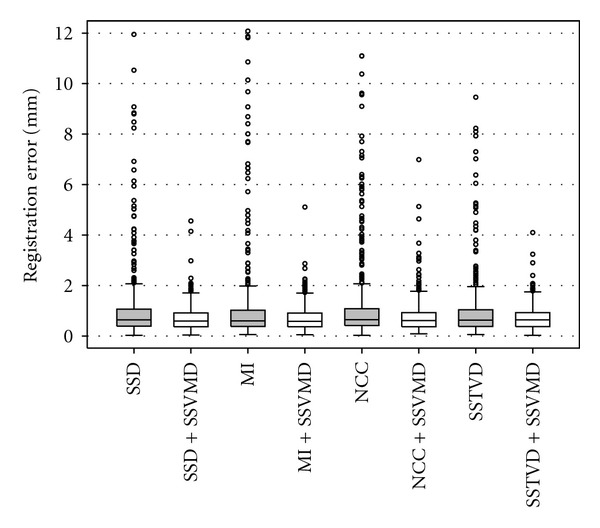
Box plot of landmark errors for six subjects after using eight registration methods. Results from methods with SSD, MI, NCC, and SSTVD metric along contain outliers beyond the error range.

 The transformation determined from registration can be used to track the landmark movements. The original average landmark error is 27.40 ± 14.37 mm with a maximum landmark error of 72.79 mm.  Table 3 shows the mean and standard deviation of landmark errors through all six subjects after using different registration methods.  Figure 6 shows the box plot of landmark errors.

### 3.4. Vessel Matching Accuracy

 The original average vessel positioning error was 12.65 ± 14.18 mm.  Table 4 shows the vessel positioning errors for six subjects after using different registration methods. The average errors and standard deviations were all decreased after adding the vesselness constraint.

**Figure 7:**
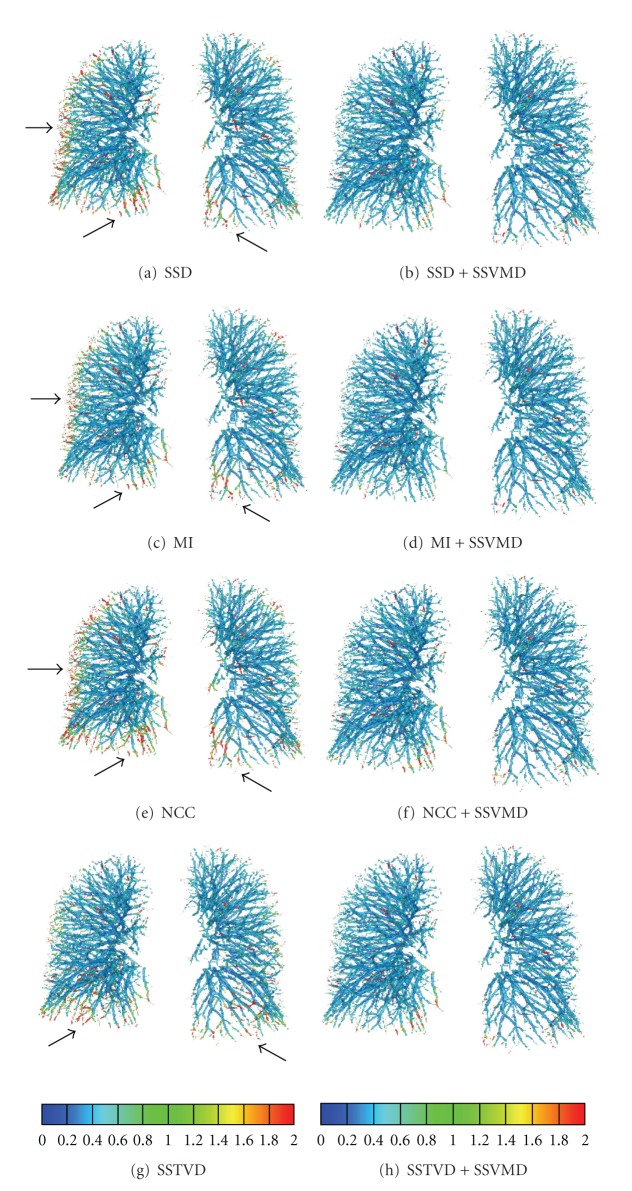
Vessel positioning errors (mm) on target vessel tree. Results are generated from eight registration methods. Arrows denote regions of large discrepancies between the deformed source and target vessel trees. Note that the errors in these regions were reduced after adding the SSVMD constraint to the registration algorithms.

Figure 7 shows the distance map on FRC vessel tree from one subject after using eight different registration methods. These results show that large errors between the deformed source and target vessel trees are reduced after adding the SSVMD constraint.

### 3.5. Fissure Alignment Distance

 For each pair of FRC and TLC images, the parenchyma regions were segmented into five lobes, and the three fissures were identified, where the lobe segmentations touched each other. The average fissure positioning error was 9.20 ± 7.94 mm before registration. The mean and standard deviation of fissure positioning error over all three fissures after using eight registration methods are shown in Table 5. The average fissure positioning error across the six subjects was 9.20 mm and decreased to around 1.50 mm and 1.00 mm without and with SSVMD cost, respectively. The fissure positioning matching accuracy improved by approximately 30% after adding SSVMD cost.

**Figure 8:**
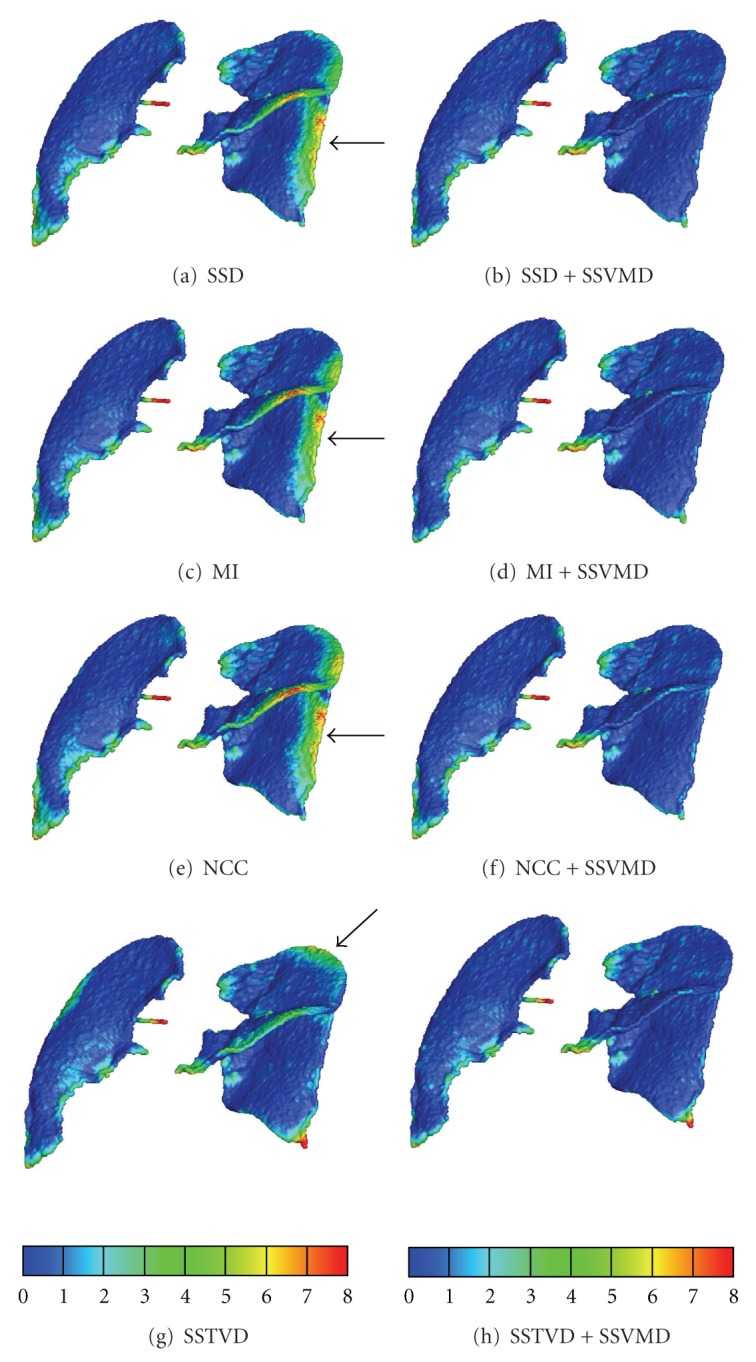
Fissure positioning error (mm) on fissure planes. Arrows denote regions of large discrepancies between the deformed source and target fissure planes.

The distance map on fissure planes from one subject after using eight different registration methods is shown in Figure 8. Notice that adding SSVMD helped improve registration accuracy in the lung regions near the thoracic cage. 

### 3.6. Assessment of Lung Function by Jacobian

**Figure 9:**
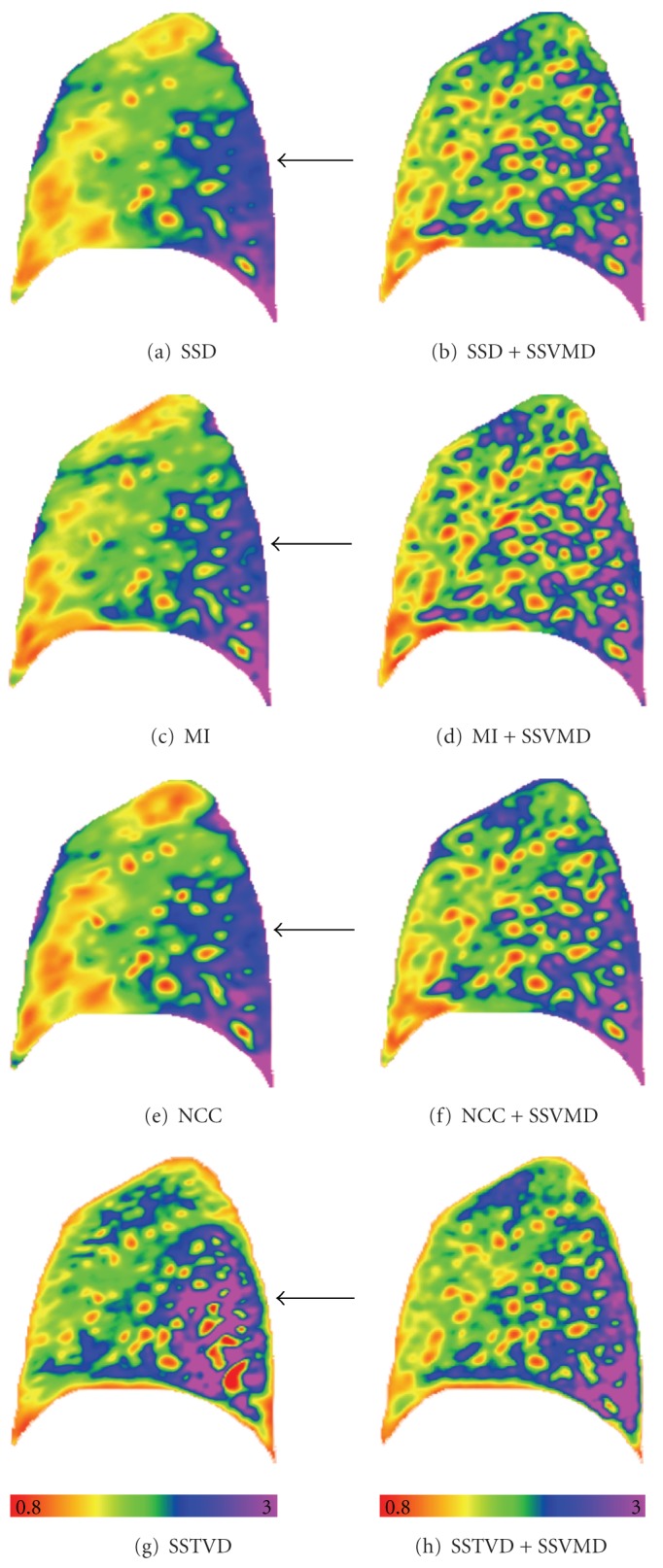
The color-coded Jacobian maps of a sagittal slice resulted from eight registration methods. Blue and purple regions have larger lung deformation, while red and orange regions are deforming less. Arrows denote regions which show different deformation patterns using intensity-only registration methods.

 Registration methods producing similar matching accuracy on the feature locations may result in different underlying parenchymal tissue functions. In order to reveal the lung tissue deformation pattern, the Jacobian of the transformation field is used to estimate the local tissue deformation [4]. The Jacobian determinant *J* at a given point gives important information about the behavior of transformation **h** near that point.  Figure 9 shows the Jacobian maps resulted from eight registration methods. Arrows denote regions that show different deformation patterns using intensity-only registration methods, but they are more similar after adding vesselness cost function.

## 4. Discussion

 The experiments presented in this paper were designed to evaluate the performance of the vesselness constraint when it was added to intensity-based registration algorithms. The experimental results of tuning weighting parameters in the total cost function suggest that using both vesselness and smoothing constraint can help minimize the similarity cost, as shown in Figure 4. Figure 5 shows that the multiresolution scheme is important and useful for solving complex registration problem efficiently. This is because the registration is first performed at a coarse scale, where the images have much fewer voxels, which are fast and can help eliminate local minima.

Table 3 and Figure 6 demonstrate that adding the SSVMD cost function reduced the mean landmark errors of the four basic registration methods. Landmarks with large errors, shown as outliers in the box plot, are aligned much better when SSVMD is used. Figure 7 reflects the fact that the SSVMD constraint helps improve matching accuracy over all four basic methods on small vessels, around lung boundaries and in the region near diaphragm. The reason for this is that blood vessels in those regions are usually small and have low intensity contrast, and thus they contribute little to conventional intensity similarity criteria. The vesselness measurement enhances blood vessel information and strengthens contribution of small vessels to registration process when using the SSVMD similarity cost. Figure 8 indicates that the SSVMD not only helps match vessel structures, but also helps improve registration accuracy in other regions, such as positions on the fissure planes near the thoracic cage.

Good matching accuracy on the feature locations does not guarantee that the parenchymal tissue is correctly aligned. Rather than evaluates the alignment accuracy, the Jacobian evaluates the quality of the transformation properties. It reveals how well the transformation preserves topology and measures the differential lung volume change. In Figure 9, the left column shows that the Jacobian maps generated by the four registration methods without SSVMD have a similar ventral to dorsal gradient as expected since the subjects were imaged in the supine orientation. However, the local tissue deformation patterns derived from these methods are different even in the methods pair SSD and SSTVD, which have similar landmark errors as shown in Table 3. This is consistent with the findings that while the intermethod variability on the landmark error is small, there may be discriminating difference in the Jacobian maps [55]. The Jacobian map from SSTVD method shows more local structure in the dorsal region. The right column shows that adding the SSVMD constraint produces Jacobian images that are much more similar across different registration methods and reveal more detailed deformation patterns especially near vessel locations. Generally, vessels have smaller volume changes comparing with parenchymal tissues during breathing cycles. The four Jacobian maps produced using registration methods with SSVMD are similar, which may imply that the derived local deformation patterns are more reliable.

Comparing the four intensity-only registration methods, registration driven by SSTVD achieved lower landmark error and lower vessel and fissure positioning errors than other three methods driven by SSD, MI, and NCC. Figures 7 and 8 reflect that SSTVD-driven registration resulted in more accurate matching within the region near the thoracic cage. The reason may be that SSTVD cost function contains a local Jacobian factor, which can constrain incorrect large displacement and help prevent distortion near the thoracic cage. After adding SSVMD on the four intensity-based cost functions, these methods generated similar transformations. SSVMD helps improve matching accuracy in regions near the thoracic cage and near the diaphragm. In our experiments, registration method using SSTVD + SSVMD resulted in the smallest matching errors. Therefore, we may consider that SSTVD + SSVMD is the best similarity cost function to register lung CT images according to our evaluation.

The effectiveness of the vesselness preserving metric was tested on a variety of lung CT data sets as part of the grand challenge “Evaluation of Methods for Pulmonary Image Registration 2010” (EMPIRE10) [56, 57]. More than 20 individual registration algorithms from different groups were applied to 30 pairs of lung CT scans in the EMPIRE10 challenge. Besides our tissue volume and vesselness preserving method, one other method called the Robust TreeReg [58] also combined intensity and feature information. The Robust TreeReg algorithm performed a robust tree registration (RTR) and added correspondences between bifurcations of the vessel tree to the voxel-based mutual information driven registration. For this registration challenge, our tissue volume and vesselness preserving method had better performance than the Robust TreeReg method. This result may imply that the vesselness measure provides more feature information than the bifurcation landmarks of the vessel tree. In addition, our tissue volume (or mass) and vesselness preserving method was shown to improve matching results compared to methods that only incorporated mass preservation [59].

## 5. Conclusions

 This paper presented nonrigid registration algorithms driven by commonly used intensity-based criteria for lung registration, a feature-based vesselness constraint, and a linear elastic smoothing constraint. Results were presented to show that adding the SSVMD constraint to existing similarity cost functions such as SSD, MI, NCC, and SSTVD reduces landmark error and improves overlap on vascular tree and fissure planes. The purpose of adding the vesselness cost in registration process is that it can help correct the mismatches of small vessels and their surrounding lung tissues. Using the SSVMD constraint was shown to produce a more detailed expansion pattern for local tissue, especially near vessel locations. Also, the expansion patterns were similar across different registration methods. This demonstrates that using the SSVMD constraint not only helps match on feature structures, but also helps align corresponding parenchymal tissues providing a more reliable pattern of local lung tissue deformation. In this paper, registration method preserving both tissue volume and vesselness measurement performed best on matching 3D lung CT data according to our evaluation.

## Figures and Tables

**Table 1:** Registration experimental parameters and cost values.

Experiment	SSVMD	REG	χ	γ	*C*_SSTVD_	*C*_SSVMD_	*C*_REG_	Jacobian min	Jacobian 1/max
CT01	No	No	0.0	0	34118	57879	172605	0.25	0.11
CT02	No	Yes	0.0	0.05	32751	49291	111770	0.38	0.12
CT03	No	Yes	0.0	0.1	32059	46156	86780	0.44	0.13
CT04	No	Yes	0.0	0.2	33571	44504	63530	0.50	0.13
CT05	No	Yes	0.0	0.4	36736	44853	43827	0.58	0.15

CT06	Yes	No	0.5	0	31288	29492	153068	0.33	0.12
CT07	Yes	Yes	0.5	0.05	30275	28611	110499	0.40	0.12
CT08	Yes	Yes	0.5	0.1	30074	27326	87990	0.44	0.13
CT09	Yes	Yes	0.5	0.2	31194	26812	63612	0.50	0.14
CT10	Yes	Yes	0.5	0.4	33426	28111	46094	0.57	0.14

CT11	Yes	No	1	0	30873	23963	161940	0.36	0.11
CT12	Yes	Yes	1	0.05	29991	22560	115510	0.40	0.11
CT13	Yes	Yes	1	0.1	29923	22328	94784	0.44	0.13
CT14	Yes	Yes	1	0.2	30956	22261	70079	0.49	0.13
CT15	Yes	Yes	1	0.4	32830	23576	50549	0.57	0.15

CT16	Yes	No	1.5	0	31713	20082	181465	0.34	0.11
CT17	Yes	Yes	1.5	0.05	31046	20059	124563	0.39	0.12
CT18	Yes	Yes	1.5	0.1	30871	20270	103340	0.43	0.12
CT19	Yes	Yes	1.5	0.2	31407	20024	76930	0.49	0.13
CT20	Yes	Yes	1.5	0.4	32982	21413	55329	0.56	0.14

CT21	Yes	No	2	0	32532	18907	192035	0.34	0.11
CT22	Yes	Yes	2	0.05	31566	18396	134317	0.39	0.12
CT23	Yes	Yes	2	0.1	31709	18742	109223	0.41	0.12
CT24	Yes	Yes	2	0.2	32172	19218	82619	0.49	0.13
CT25	Yes	Yes	2	0.4	33252	19946	59525	0.56	0.14

**Table 2:** An example multiresolution scheme.

Image resolution	B-spline grid size	Max. iteration
1/8	128 mm	200
	64 mm	200

1/4	32 mm	200
	16 mm	200

1/2	16 mm	50
	8 mm	50

1	8 mm	20
	4 mm	20

**Table 3:** Landmark errors (mm) using six subjects.

	Without SSVMD		With SSVMD	
	Avg.	Max	Avg.	Max
SSD	0.95 ± 1.29	15.97	0.71 ± 0.46	4.56
MI	1.05 ± 1.85	16.72	0.68 ± 0.46	5.11
NCC	1.07 ± 1.57	15.18	0.74 ± 0.57	6.99
SSTVD	0.92 ± 1.28	15.38	0.71 ± 0.45	4.1

**Table 4:** Vessel positioning errors (mm) averaged over six subjects.

	SSD	MI	NCC	SSTVD
Without SSVMD	0.74 ± 0.62	0.70 ± 0.61	0.74 ± 0.65	0.67 ± 0.57
With SSVMD	0.63 ± 0.52	0.64 ± 0.53	0.65 ± 0.54	0.60 ± 0.49

**Table 5:** Fissure positioning errors (mm) using six subjects.

	SSD	MI	NCC	SSTVD
Without SSVMD	1.48 ± 1.84	1.48 ± 1.78	1.62 ± 2.04	1.41 ± 1.61
With SSVMD	1.05 ± 0.82	1.05 ± 0.79	1.07 ± 0.89	1.00 ± 0.69
